# Myocardial perfusion MRI with SW-CG-HYPR: a comparison to conventional SR-Turbo-FLASH and x-ray angiography in patients with suspected coronary artery disease

**DOI:** 10.1186/1532-429X-14-S1-P303

**Published:** 2012-02-01

**Authors:** Heng Ma, Jun Yang, Jing Liu, Lan Ge, David Chen, Kai Lin, Jing An, Lixin Jin, Kuncheng Li, Debiao Li

**Affiliations:** 1 Radiology, Yuhuangding Hospital, Yantai, China; 2 Xuanwu Hospital, Capital Medical University, Beijing, China; 3 University of California, Los Angeles, CA, USA; 4 Siemens Mindit Magnetic Resonance, Shenzhen, China; 5 Siemens Limited China, Shanghai, China

## Summary

In the current study, we prospectively compared the diagnostic value of SW-CG-HYPR and conventional SR-Turbo-FLASH for myocardial perfusion MRI in patients with suspected CAD. Compared with conventional SR-Turbo-FLASH, SW-CG-HYPR allows increased spatial coverage (whole left ventricular coverage), improved temporal and spatial resolution and SNR, reduced motion artifacts and has higher diagnostic accuracy in patients with suspected CAD.

## Background

A sliding-window (SW) conjugate-gradient (CG) highly constrained back-projection reconstruction (HYPR) (SW-CG-HYPR) technique has been developed for time-resolved myocardial perfusion imaging. Using this method, the acquisition time per cardiac cycle can be reduced dramatically while maintaining the temporal resolution of one frame per heartbeat in myocardial perfusion MRI, allowing increased spatial coverage (whole left ventricular coverage), improved temporal and spatial resolution and SNR, and reduced motion artifacts compared with the conventional SR-Turbo-FLASH sequence. However, the diagnostic accuracy of myocardial perfusion MRI with SW-CG-HYPR for detecting coronary artery disease (CAD) has not been directly compared to that with conventional SR-Turbo-FLASH. The purpose of this study was to prospectively compare the diagnostic value of SW-CG-HYPR and conventional SR-Turbo-FLASH for myocardial perfusion MRI in patients with suspected CAD.

## Methods

Thirty consecutive patients (17 men, 13 women; age range, 39-74 years; mean age, 55 years ± 19) with suspected CAD who were scheduled for coronary angiography underwent myocardial perfusion MRI with both SW-CG-HYPR and SR-Turbo-FLASH at 3.0T. Perfusion defects were interpreted visually by 2 blinded observers and were correlated to x-ray angiographic stenoses ≥ 50%. Receiver-operating characteristic (ROC) curve analysis was used to compare the diagnostic performance of the two imaging techniques.

## Results

The prevalence of CAD was 60%. Compared with SR-Turbo-FLASH, SW-CG-HYPR produced better left ventricular (LV) coverage (whole LV vs. only 3 slices). In the per-patient analysis, SW-CG-HYPR provided a higher sensitivity (94% vs. 89%), specificity (83% vs. 75%) and diagnostic accuracy (90% vs. 83%) for the detection of CAD than SR-Turbo-FLASH. In the per-vessel analysis, the diagnostic performance of SW-CG-HYPR was significantly greater than that of SR-Turbo-FLASH for the overall detection of CAD (area under ROC curve: 0.96 ± 0.02 vs. 0.90 ± 0.03, respectively; p < 0.05).

## Conclusions

High-resolution, whole left ventricle myocardial perfusion MRI with SW-CG-HYPR allows higher diagnostic accuracy than conventional SR-Turbo-FLASH in patients with suspected CAD.

## Funding

This work was supported by National Institute of Health (NIBIB EB002623) and National Natural Science Foundation of China (30828009).

